# Alexithymia in schizophrenia spectrum disorders and dissociative disorders: two meta-analytic reviews

**DOI:** 10.1038/s41537-026-00765-8

**Published:** 2026-05-14

**Authors:** Bryan Ho-wang Yu, Joyce Ho, Suzanne Ho-wai So

**Affiliations:** Department of Psychology, The Chinese University of Hong Kong, Hong Kong SAR, China

**Keywords:** Human behaviour, Schizophrenia, Emotion

## Abstract

Schizophrenia-spectrum disorders (SSDs) and dissociative disorders (DDs) often co-occur, and both show emotion processing deficits. Alexithymia—comprising difficulty identifying feelings (DIF), difficulty describing feelings (DDF), and externally oriented thinking (EOT)—is considered a shared vulnerability factor, yet the relative prominence of each facet within and across these disorders remains uncertain. The current two meta-analyses synthesised severity of overall alexithymia and its facets in SSDs (Meta-analysis 1) or DDs (Meta-analysis 2) compared to psychiatrically healthy controls. A preregistered search of PsycINFO, PubMed, and Embase (1970–31 December 2024) identified adult case-control studies. Pooled Hedges’ *g* values were estimated with random-effects meta-analysis models, then compared across diagnoses (moderator analyses) with mixed-effects meta-analysis models. Meta-analysis 1 pooled 27 SSD studies (1477 patients, 1249 controls). Meta-analysis 2 pooled 30 DD studies (842 patients, 796 controls). Both patient groups scored higher than controls on overall alexithymia and all facets. In SSDs, DIF showed the largest group effect (*g* = 0.894; CI 0.667–1.122), whereas DDF (*g* = 0.622; CI 0.468–0.775) and EOT (*g* = 0.585; CI 0.400–0.770) showed moderate effects. In DDs, both DIF (*g* = 1.311; CI 0.991–1.632) and DDF (*g* = 0.954; CI 0.707–1.202) showed large effects, whereas EOT (*g* = 0.427; CI 0.163–0.690) showed a small-to-moderate effect. Between the disorder groups, after removing extreme effects, DDs exhibited greater DIF and DDF than SSDs, whereas SSDs showed higher EOT. Heterogeneity was moderate-to-high, and publication bias was possible in SSD studies. Alexithymia is a significant transdiagnostic deficit, but its facet profiles differ between SSDs and DDs. Emotion-focused interventions should therefore target facet-specific deficits prominent to each disorder group.

## Introduction

### Alexithymia

Nemiah and Sifneos first described alexithymia as a trait marked by an inability to recognise or articulate feelings^1,2^. It is anchored in three facets: difficulty identifying feelings (DIF), difficulty describing feelings (DDF), and externally oriented thinking (EOT)—a cognitive tendency to focus on the external world while neglecting one’s own internal states. Other proposed facets, such as restricted imagination/fantasy related to drives and feelings^3^ or difficulties emotionalising^4^, lack internal reliability and show poor psychometric properties^5–7^. Therefore, the three-factor structure remains the prevailing theoretical framework. Alexithymia is often implicated in psychopathology^8–10^, with facet-level analyses suggesting that different disorders may exhibit distinct alexithymic profiles and consequently, unique deficits in emotional functioning^11,12^.

### Alexithymia in schizophrenia spectrum disorders

Schizophrenia Spectrum Disorders (SSDs) encompass schizophrenia, schizoaffective disorder, delusional disorder, and related diagnoses. Besides positive symptoms like hallucinations and delusions, patients often experience negative symptoms such as blunted affect and anhedonia^13^. Patients with SSDs show relatively preserved internal emotional experience despite diminished emotional expressiveness^14^, a discrepancy that alexithymia may help to explain because higher alexithymia is associated with increased negative symptoms and habitual suppression of emotional display in individuals with schizophrenia^15,16^.

A meta-analysis of eight case-control studies reported higher overall alexithymia in SSDs than healthy controls^17^; however, seven of these studies focused exclusively on schizophrenia, and the pooled effect showed substantial heterogeneity. This heterogeneity may reflect subgroup differences (e.g., higher alexithymia in patients with prominent negative symptoms^18^) or unmodelled variability at the facet level—which the meta-analysis did not examine. A few other studies have compared facet-specific difficulties across psychiatric disorders, yielding inconsistent findings: Son et al.^19^ found that psychotic patients scored higher than patients with depression, anxiety, or somatoform disorders on the DDF subscale of the Toronto Alexithymia Scale (TAS-20)^20^, whereas Yıldırım et al.^21^, using similar diagnostic groups, reported higher overall alexithymia and DIF in psychotic patients but no DDF difference. Taken together, existing evidence on alexithymia in SSDs is largely limited to overall-level comparisons with restricted generalisability, and facet-level findings remain inconclusive.

### Alexithymia in dissociative disorders

Dissociative disorders (DDs) include primarily dissociative identity disorder, dissociative amnesia, depersonalisation-derealisation disorder, and dissociative neurological symptom disorder (formerly conversion disorder)^22^. DDs can be broadly conceptualised through compartmentalisation or detachment phenomena^23–25^: compartmentalisation denotes involuntary loss of control over memory, sensation, or motor function (e.g., dissociative amnesia, non-epileptic seizure, functional motor symptoms), while detachment describes the altered state of consciousness characterised by a sense of separation from the self or world (e.g., depersonalisation, derealisation, emotional numbing). Both reflect a disconnection within self-experiences and are often viewed as a defence against extreme stress or trauma^26^.

Dissociation disrupts emotion-cognition integration and is closely related to alexithymia^27–29^. A meta-analysis of 19 studies drawing on mixed psychiatric and non-clinical samples reported a positive association between alexithymia and dissociation, but alexithymia severity within DDs was not specifically addressed^29^. Case-control studies have generally found higher overall alexithymia in dissociative patients than in healthy individuals^30,31^. However, facet-level findings diverge, with some studies reporting higher DIF and DDF but not EOT^30,31^, and others reporting elevations in all three facets^32,33^. Additionally, as most studies focus on single dissociative diagnoses only, it is unclear whether alexithymia differs between detachment and compartmentalisation DDs.

### Alexithymic profiles between the two disorder clusters

Despite being distinct diagnoses, comorbidity between SSDs and DDs is common. Among patients with SSDs, 9–50% met criteria for a DD, while 27–41% of those with DDs met criteria for a comorbid psychotic disorder^34–36^. The two disorder clusters also share clinical phenomena such as delusions of control, auditory hallucinations, depersonalisation, and blunted affect^37–39^. Because alexithymia is linked to affective restriction^40,41^ and is present in both clusters, a direct facet-level comparison of their alexithymic profiles could clarify shared versus disorder-specific affective processing deficits and guide tailored interventions. Yet, no study has directly contrasted SSDs and DDs on alexithymia, and the relative prominence of each facet remains unknown.

### Objective

To delineate the alexithymic profiles of SSDs and DDs, two parallel meta-analyses were conducted. Meta-analysis 1 synthesised studies comparing patients with SSDs against healthy controls. Meta-analysis 2 synthesised studies comparing patients with DDs against healthy controls. For each analysis we extracted effect sizes of mean differences for overall alexithymia and for DIF, DDF, and EOT respectively. The aggregated effects were then compared to identify the dominant facets within each disorder cluster and to contrast their magnitude between clusters.

## Methods

**Fig. 1:**
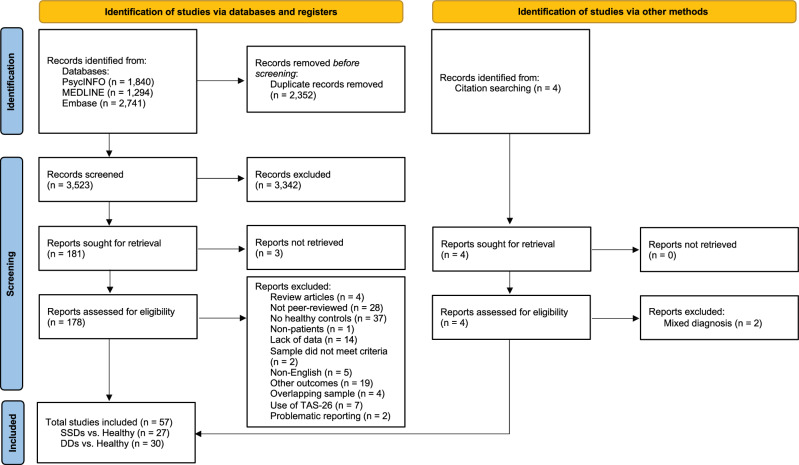
Flowchart for the identification and selection of eligible studies.

The review was prospectively registered on PROSPERO (CRD42024502519) and conducted in line with PRISMA guidelines^42^. Deviations from the registered protocol were: 1) the R package ‘meta’^43^ was used for greater versatility for graphical output; and 2) a subgroup analysis was added to Meta‑analysis 1. Figure 1 presents the PRISMA flowchart. The full checklist is in Supplementary Material S1.

### Eligibility criteria

We included English-language, peer-reviewed studies that compared alexithymia in adults (18–65 years) with an SSD or a DD against psychiatrically healthy controls. Because the focus was on disorder clusters, all SSD diagnoses were eligible for Meta-analysis 1 and all DD diagnoses were eligible for Meta-analysis 2. Acceptable designs were observational case-control studies. Baseline data from longitudinal or experimental work were also included. Eligible alexithymia measures captured all three core facets (DIF, DDF, EOT), such as the Toronto Alexithymia Scale-20 (TAS-20)^20^ and the cognitive dimension of the Bermond-Vorst Alexithymia Questionnaire (BVAQ)^4^. (The BVAQ ‘Analysing’ subscale conceptually maps onto the TAS-20 ‘EOT’ subscale. Vorst and Bermond^4^ described the difference as nominal, and Preece et al.^7^ showed through factor analysis that both load onto the same latent factor). The 26-item version of the TAS^3^ was excluded because several items cross-loaded on multiple factors^20^.

Studies were excluded if they: (i) used a mixed clinical group; (ii) lacked sufficient data for the computation of effect sizes or the data could not be obtained; and (iii) were case reports, review papers, commentaries and editorial letters, book chapters, protocols, dissertations or abstracts.

### Search strategy

A systematic search of the electronic databases PsycINFO, MEDLINE, and Embase was conducted, limited to publications from 1970 through 31 December 2024. The following search terms were used: (schizo* OR “psychotic disorder*” OR psychos*s [for Meta-analysis 1]) OR (“dissociative disorder*” OR depersonali?ation OR dereali?ation OR “dissociative amnesia” OR “dissociative identit*” OR “multiple personalit*” OR fugue OR dissociation OR detachment OR compartmentali?ation OR “conversion disorder*” OR “functional neurologic*” OR psychogenic OR “non-epileptic” OR “dissociative seizure*” OR “functional motor*” [for Meta-analysis 2]) AND (alexithymia OR alexithymic OR “identify* emotions” OR “identify* feelings” OR “describ* emotions” OR “describ* feelings” OR “externally orient* thinking*” OR “emotion* recognition” OR “emotion* differentiation” OR “emotion* awareness” OR “emotion* granularity” OR “emotion* clarity”). Reference lists of included studies as well as relevant review papers were also looked up to identify any additional papers not captured in the database search^17,29,44^.

### Selection of studies

The lead author (BHY) screened all titles and abstracts and reviewed full-text of all eligible studies, and the second author (JH) and a trained co-rater (KS) independently double-screened 20% of titles and abstracts. Any disagreement was resolved by discussion among the three co-raters.

### Data extraction

BHY recorded all study information in Excel. JH and KS independently re-extracted data from 20% of the papers to verify accuracy. Extracted data include study design, sample sizes and characteristics (gender, age, diagnosis), matched demographic variables, measures of alexithymia and means and standard deviations (SD). When only medians and ranges were provided, means and SDs were estimated using Wan et al.’s method^45^. If descriptive statistics were missing, test statistics or published effect sizes were used. Study authors were contacted for any remaining data.

### Quality assessment

Study quality was appraised with an adapted checklist based on the Agency for Healthcare Research and Quality^46^ and the Cochrane Risk of Bias tool^47^. Four domains were evaluated: selection, detection, attrition, and reporting bias (see Supplementary Material S2). Each study was rated as ‘Good’ (low risk), ‘Fair’, or ‘Poor’ (high risk). BHY rated every study, and JH and KS independently reviewed 20% of the sample. Any disagreement was resolved by discussion among the three co-raters.

### Data analysis

For each study, Hedges’ *g*, its 95% confidence interval (CI) and the associated *z*- and two-sided *p* values were computed for overall alexithymia and for each facet (DIF, DDF, EOT). When a paper reported more than one patient subgroup (either multiple subgroups of the same diagnosis or different diagnoses within the same cluster), subgroup data were pooled to yield a single effect size^48^. If an overall alexithymia score was unavailable, it was estimated from pooled subscale means^48^. Two random-effects meta-analyses were then run using the R package ‘meta’^43^, with variance estimated using the restricted maximum-likelihood (REML) estimator. The first compared patients with any SSDs against healthy controls and the second compared patients with any DDs against healthy controls. Within each cluster, post-hoc tests contrasted aggregated effects for DIF, DDF, and EOT to evaluate their relative strength. Subsequently, moderator analyses using mixed-effects models were conducted to test whether effects for overall alexithymia and each facet differ between clusters of SSDs and DDs. At least five effect sizes per group was required^48^.

Heterogeneity was assessed using the *Q*-statistics and *I*^2^ indices, interpreted as none (0%), low (25%), moderate (50%), and high (75%)^49^. We tested whether measurement instrument influences effect sizes via subgroup analyses. Sensitivity analyses excluded effect sizes more than two SDs from the mean. Publication bias was evaluated with funnel-plot inspection, Egger’s regression, and the Begg-Mazumdar rank-correlation test.

### Additional analysis

Subgroup analyses using mixed-effects models were performed to test variability within each diagnostic cluster. In Meta-analysis 1 (SSDs vs. healthy controls), studies that focused solely on schizophrenia were contrasted with those that combined several SSD diagnoses. In Meta-analysis 2 (DDs vs. healthy controls), effects were compared between detachment (depersonalisation disorder) and compartmentalisation (conversion disorder, psychogenic non-epileptic seizures [PNES], and functional motor disorder [FMD]).

### Ethics

A statement of ethics is not applicable because this study is based exclusively on published literature.

## Results

Full references of all included studies are listed in Supplementary Material S3.

### Meta-analysis 1 results

Meta-analysis 1 included 27 studies comparing patients with any SSDs to healthy controls, totalling 2726 participants (1477 patients and 1249 controls). Four studies divided the clinical group into subcategories: authors of two studies^50,51^ supplied mean and SD for the full clinical group on request; for the other two studies^18,52^, subgroup data were pooled for analysis. Four studies^53–56^ did not report overall alexithymia scores; therefore, they were estimated from pooled subscale means. In total, 27 effect sizes were included in the meta-analysis of overall alexithymia. Fewer studies reported subscores, yielding 19 effect sizes each in the meta-analysis of DIF and DDF, and 17 effect sizes for EOT.

#### Study characteristics

Table 1a outlines key study characteristics. All studies were observational case-control comparisons of adults diagnosed with an SSD. Twenty-two studies excluded patients with alcohol or substance dependence, and 13 excluded those with intellectual impairment. Most samples consisted of chronic, clinically stable patients (*k* = 22); others were comprised of first‑episode schizophrenia (*k* = 1), active or acute‑phase psychosis (*k* = 2), and unclear illness stage (*k* = 2). Samples included both inpatients and outpatients, and antipsychotic treatment was reported in 20 studies. Alexithymia was assessed predominantly with the TAS-20 (*k* = 22); five studies used the BVAQ.

**Table 1:** Characteristics of all included studies.

Study, country	Design	Case			Control		Matched variables	Measure of alexithymia	Diagnostic tool	Risk of bias
		Sample characteristics	*N*; % female	Mean age (SD)	*N*; % female	Mean age (SD)				
(a) Studies included in Meta-analysis 1 (*k* = 27)										
Cedro et al. (2001), Poland	Cross-sectional	Paranoid SZ (Remitted, receiving anti-psychotics)	50; 50%	42.3 (11.0)	50; 50%	42.1 (10.8)	Age, gender, years of education	TAS-20	DSM-IV	Selection: Fair Detection: Fair Attrition: Good Reporting: Good
Etchepare et al.^50^ (2019), France	Cross-sectional	SZ or SZA (Clinically stable, receiving anti-psychotics)	94; 21.3%	36.7 (10.3)	120; 55%	36.5 (12.9)	Age	BVAQ	DSM-5	Selection: Good Detection: Good Attrition: Good Reporting: Good
He et al. (2022), China	Cross-sectional	SZ (First-episode)	107; 51.4%	36.94 (10.73)	45; 48.9%	32.47 (10.94)	Age, gender education, marital and employment status	TAS-20	MINI 6.0.0, DSM-5	Selection: Good Detection: Good Attrition: Good Reporting: Good
Herbert et al.^52^ (2018), Germany	Cross-sectional	SZ or SZA (Clinically stable, receiving anti-psychotics)	21; 38.1%	34.42 (9.83)	21; 38.1%	33.52 (10.30)	Age, gender	TAS-20	SCID-IV	Selection: Good Detection: Good Attrition: Good Reporting: Fair
Hyatt et al.^53^ (2022), USA	Cross-sectional	SZ (Clinically stable, receiving anti-psychotics)	41; 29.3%	30.9 (3.8)	27; 49%	29.1 (3.6)	Controlled for age, IQ and gender	BVAQ	DSM-5	Selection: Good Detection: Good Attrition: Good Reporting: Good
Kamburidis (2024), Bulgaria	Cross-sectional	SZ	30; 26.7%	20-64	39; 84.6%	20–64	None	TAS-20	ICD-10	Selection: Poor Detection: Fair Attrition: Good Reporting: Poor
Kimhy et al.^55^ (2012), USA	Cross-sectional	SSDs (Active psychosis, receiving anti-psychotics)	44; 36%	30.33 (8.08)	20; 50%	24.20 (4.62)	Gender, ethnicity	TAS-20	DIGS	Selection: Good Detection: Good Attrition: Good Reporting: Good
Kimhy et al.^54^ (2016), USA	Cross-sectional	SZ (Chronic cases, receiving anti-psychotics)	87; 37%	33.45 (9.47)	50; 48%	23.04 (4.10)	Gender, ethnicity, race	TAS-20	DIGS	Selection: Good Detection: Good Attrition: Good Reporting: Good
Kubota et al.^77^ (2011), Japan	Cross-sectional	SZ (Chronic cases, receiving anti-psychotics)	21; 33.3%	35.3 (9.4)	24; 33.3%	37.4 (11.5)	Age, gender, years of education	TAS-20	SCID-IV	Selection: Good Detection: Good Attrition: Good Reporting: Good
Kubota et al. (2012), Japan	Cross-sectional	SZ (Chronic cases, receiving anti-psychotics)	44; 41%	36.3 (10.1)	44; 41%	34.4 (12.4)	Age, gender, IQ	TAS-20	SCID-IV	Selection: Good Detection: Good Attrition: Good Reporting: Good
Kumar et al.^59^ (2018), India	Cross-sectional	SZ (Clinically stable, outpatients)	30	39.63 (8.95)	30	40.33 (8.73)	Age, marital status, years of education	TAS-20	ICD-10	Selection: Fair Detection: Good Attrition: Good Reporting: Good
Lee et al.^57^ (2021), Korea	Cross-sectional	SZ (Chronic cases, receiving antipsychotics)	22; 55%	33.8 (8.6)	22; 55%	33.7 (8.2)	Age, gender	TAS-20	Not provided	Selection: Poor Detection: Fair Attrition: Good Reporting: Fair
Luo et al. (2021), China	Cross-sectional	SZ (Chronic cases)	135; 39%	43.45 (11.72)	73; 67%	40.35 (9.96)	Age, years of education	TAS-20	DSM-5	Selection: Good Detection: Good Attrition: Good Reporting: Good
Opoka et al. (2021), Germany	Cross-sectional	SSDs (In- and out-patients, 95% receiving anti-psychotics)	60; 63.3%	40.15 (11.66)	40; 67.5%	40.03 (10.78)	Age, gender, years of education	TAS-20	MINI	Selection: Good Detection: Good Attrition: Good Reporting: Fair
Ospina et al. (2019), USA	Cross-sectional	SZ or SZA (Chronic cases)	45; 46.7%	44.51 (12.40)	50; 58%	38.06 (12.84)	Gender, ethnicity	TAS-20	SCID-IV	Selection: Good Detection: Good Attrition: Good Reporting: Good
Raugh et al.^60^ (2024), Georgia	Cross-sectional	SZ (Chronic cases, outpatients)	22; 63.6%	41.27 (10.6)	55; 69.1%	39.07 (10.62)	Age, gender, ethnicity	TAS-20	SCID-IV	Selection: Good Detection: Good Attrition: Good Reporting: Fair
Regenbogen et al. (2015), Germany	Cross-sectional	Paranoid SZ (In- and out-patients, receiving anti-psychotics)	19	37.30 (8.44)	24	35.25 (9.80)	Age, years of education	TAS-20	SCID-IV	Selection: Good Detection: Good Attrition: Good Reporting: Fair
Swart et al. (2013), The Netherlands	Cross-sectional	SZ (In- and out-patients, 90% receiving anti-psychotics)	18; 16.7%	29.44 (5.81)	18; 33.3%	28.44 (8.09)	Age, gender, educational level	BVAQ	SCAN-2.1, DSM-IV	Selection: Good Detection: Good Attrition: Good Reporting: Good
Tang et al.^18^ (2016), China	Cross-sectional	SZ (Chronic cases, receiving anti-psychotics)	94; male only	47.6 (6.4)	54; male only	47.6 (10.1)	Age	TAS-20	SCID-IV	Selection: Good Detection: Good Attrition: Good Reporting: Good
Torregrossa et al. (2022), USA	Cross-sectional	SZ or SA (Chronic cases, receiving anti-psychotics)	30; 46.7%	47.93 (10.21)	28; 50%	48.68 (8.2)	Age, gender	TAS-20	DSM-5	Selection: Good Detection: Good Attrition: Good Reporting: Good
Trémeau et al. (2013), USA	Cross-sectional	SZ (Chronic cases, inpatients, receiving anti-psychotics)	86; 21%	38.83 (11.78)	45; 27%	36.36 (13.01)	Age, gender, ethnicity	TAS-20	SCID-IV	Selection: Good Detection: Good Attrition: Good Reporting: Fair
Vakhrusheva et al. (2020), USA	Cross-sectional	SSDs	53; 40%	32.00 (8.30)	19; 63%	22.63 (3.37)	Gender, ethnicity	TAS-20	DSM-IV, DIGS	Selection: Good Detection: Good Attrition: Good Reporting: Fair
Valdés et al. (2018), Spain	Cross-sectional	Paranoid SZ (Receiving anti-psychotics)	37; 32.5%	28.68 (5.17)	37; 35.2%	28.17 (5.06)	Age, gender, socio-economic status	TAS-20	SCID-IV-TR	Selection: Good Detection: Good Attrition: Good Reporting: Good
van der Velde, et al.^58^ (2015), The Netherlands	Cross-sectional	SZ (Clinically stable, receiving anti-psychotics)	38; 24%	34.4 (10.6)	109; 50%	31.4 (10.4)	Controlled for age, gender, education level	BVAQ	MINI-plus	Selection: Fair Detection: Fair Attrition: Good Reporting: Good
van ‘t Wout et al.^51^ (2007), The Netherlands	Cross-sectional	SSDs (Clinically stable, in- and out-patients, 91% receiving anti-psychotics)	43; 44%	31.14 (7.30)	44; 34%	31.98 (9.16)	Age, gender, years of education	BVAQ	CASH, MINI-plus	Selection: Good Detection: Good Attrition: Good Reporting: Good
Yu et al.^56^ (2011), China	Cross-sectional	Paranoid SZ (Acute, 60% first episode, receiving anti-psychotics)	60; 50%	25.85 (9.10)	60; 55%	23.17 (7.58)	Age, gender, education level	TAS-20	ICD-10	Selection: Fair Detection: Good Attrition: Good Reporting: Good
Zou et al. (2018), China	Cross-sectional	SZ (Outpatients, receiving anti-psychotics)	146; 56.2%	36.29 (10.30)	73; 49.3%	35.95 (10.63)	Age, gender, IQ, years of education	TAS-20	SCID-IV	Selection: Good Detection: Good Attrition: Good Reporting: Good
(b) Studies included in Meta-analysis 2 (*k* = 30)										
Bewley et al. (2005), UK	Cross-sectional	PNES (Outpatients)	21; 85.7%	40.29 (15.31)	21; 85.7%	40.17 (15.64)	Age, gender	TAS-20	EEG recordings, medical records	Selection: Good Detection: Good Attrition: Good Reporting: Good
Daniels et al. (2015), Germany	Cross-sectional	DPD (Chronic cases, 36% on psychotropic medication)	25; 72.0%	31.60 (8.09)	23; 78.3%	29.96 (7.99)	Age, gender	TAS-20	SCID-D, SCID-IV, ICD-10	Selection: Good Detection: Good Attrition: Good Reporting: Fair
del Río-Casanova et al.^65^ (2018), Spain	Cross-sectional	CD (Outpatients)	43; 88.4%	44.81 (3.20)	42; 85.8%	41.99 (3.68)	Age, gender	TAS-20	DSM-5	Selection: Good Detection: Good Attrition: Good Reporting: Fair
Demartini et al. (2014), UK	Cross-sectional	FMS (Outpatients)	55; 76%	43.00 (10.55)	34; 68%	42.18 (11.32)	Age, gender	TAS-20	DSM-IV, Fahn & Williams criteria, medical notes	Selection: Good Detection: Good Attrition: Good Reporting: Good
Demartini et al. (2016a), UK	Cross-sectional	PNES, FMD (Outpatients, current symptoms)	PNES: 20; 75% FMD: 20; 85%	PNES: 45.9 (14.8) FMD: 45.7 (15.8)	20; 80%	43.1 (17.0)	Age, gender	TAS-20	PNES: video-EEG, LaFrance criteria FMD: Fahn & Williams criteria	Selection: Good Detection: Good Attrition: Good Reporting: Fair
Demartini et al. (2016b), UK	Cross-sectional	FMS (Chronic cases, outpatients)	16; 81%	40.68 (11.45)	18; 72%	37.22 (9.46)	Age, gender	TAS-20	DSM-5, Fahn & Williams criteria	Selection: Poor Detection: Good Attrition: Good Reporting: Good
Demartini et al. (2017), Italy	Cross-sectional	FMS (Current symptoms)	20; 70%	45.75 (15.87)	20; 80%	42.10 (13.34)	Gender	TAS-20	SCID-IV, Fahn & Williams criteria	Selection: Good Detection: Good Attrition: Good Reporting: Fair
Demartini et al. (2019), Italy	Cross-sectional	FMS (Current symptoms)	13; 84.6%	49.7 (17.1)	14; 92.9%	45.6 (15.9)	Age, gender	TAS-20	Fahn & Williams criteria	Selection: Fair Detection: Good Attrition: Good Reporting: Fair
Güleç et al. (2014), Turkey	Cross-sectional	CD (Current symptoms)	94; 85%	30.54 (10.79)	50; 70%	34.64 (11.94)	Age, gender, educational level	TAS-20	SCID-IV	Selection: Good Detection: Good Attrition: Good Reporting: Good
Gulpek et al.^30^ (2014), Turkey	Cross-sectional	CD (Chronic cases, outpatients)	47; 78.7%	32.34 (1.00)	46; 78.3%	32.61 (1.03)	Age, gender, educational level	TAS-20	SCID-IV	Selection: Good Detection: Good Attrition: Good Reporting: Good
Gürsoy et al. (2021), Turkey	Cross-sectional	PNES (Outpatients)	28; 78.6%	36.86 (8.39)	28; 71.4%	35.11 (7.19)	Age, gender, educational level	TAS-20	SCID-IV, video-EEG, medical notes	Selection: Good Detection: Good Attrition: Good Reporting: Good
Herrero et al. (2020), France	Cross-sectional	PNES (Current symptoms)	34; 100%	34.4	34; 100%	35.0	Age, educational level	TAS-20	video-EEG, MINI	Selection: Fair Detection: Good Attrition: Good Reporting: Good
Jalilianhasanpour et al. (2018), USA	Cross-sectional	FND (Current symptoms)	50; 74%	40.8 (12.5)	47; 72.3%	37.7 (11.8)	Age, gender, education status, ethnicity	TAS-20	PNES: LaFrance criteria FMD: Fahn & Williams criteria	Selection: Good Detection: Good Attrition: Good Reporting: Fair
Jungilligens et al.^33^ (2020), Germany	Cross-sectional	PNES (Current symptoms)	20; 70%	32.9 (12.8)	20; 70%	29.4 (9.9)	Age, gender	TAS-20	video-EEG, MINI	Selection: Fair Detection: Good Attrition: Good Reporting: Good
Jungilligens et al. (2021), Germany	Cross-sectional	PNES (Current symptoms)	20; 75%	34.9 (11.2)	20; 75%	36.6 (12.2)	Age, gender, years of education	TAS-20	video-EEG, LaFrance criteria, MINI	Selection: Good Detection: Good Attrition: Good Reporting: Good
Lemche et al.^67^ (2013), UK	Cross-sectional	DPD (Current symptoms, 33% receiving psychotropic medication)	9; 44%	36.11 (7.31)	12; 42%	27.25 (4.95)	Gender, educational level, socio-economic status	TAS-20	DSM-IV-TR	Selection: Poor Detection: Good Attrition: Good Reporting: Good
Marotta et al.^66^ (2020), Italy	Cross-sectional	FMD	25; 72%	42.80 (14.29)	25; 64%	39.80 (16.96)	Age, gender	TAS-20 (FMD: 23 completed)	Fahn & Williams / Gupta & Lang criteria	Selection: Fair Detection: Good Attrition: Good Reporting: Good
Millman et al. (2024), UK	Cross-sectional	FND (Current symptoms)	17	18-65	17	18-65	Age, gender, ethnicity	TAS-20	DSM-5, medical record	Selection: Good Detection: Good Attrition: Good Reporting: Good
Monde et al. (2013), USA	Cross-sectional	DPD (Chronic cases, 43% receiving psychotropic medication)	14; 29%	30.8 (7.2)	14; 36%	31.3 (11.9)	Age, gender, years of education	TAS-20	SCID-D, SCID-IV	Selection: Fair Detection: Good Attrition: Good Reporting: Fair
Nisticò et al. (2024), Italy	Cross-sectional	FMD (Current symptoms)	10; 60.0%	50.4 (15.5)	11; 63.6%	46.0 (16.7)	Age, gender	TAS-20	DSM-5, Gupta & Lang diagnostic criteria	Selection: Good Detection: Good Attrition: Good Reporting: Good
O’Brien et al. (2015), Ireland	Cross-sectional	PNES (Chronic cases)	19; 68%	30.0 (8.8)	19; 68%	29.7 (7.0)	Age, gender	TAS-20	video-EEG, MRI, SCID, LaFrance criteria	Selection: Good Detection: Good Attrition: Good Reporting: Fair
Pick et al. (2023), UK	Cross-sectional	FND (Current symptoms)	16; 75%	36.1 (10.8)	17; 76%	39.0 (11.0)	Age, gender, years of education	TAS-20	SCID-V-RV, medical records	Selection: Good Detection: Good Attrition: Good Reporting: Fair
Poli et al.^32^ (2022), Italy	Cross-sectional	PNES (Current symptoms)	14; 79%	43.64 (11.76)	16; 69%	44.69 (12.74)	Age, gender	TAS-20	Video-EEG, LaFrance criteria	Selection: Fair Detection: Good Attrition: Good Reporting: Good
Ricciardi et al. (2021), UK	Cross-sectional	FMD (Chronic cases)	22; 86%	44.8 (14.8)	23; 61%	41.5 (15.0)	Age, gender	TAS-20	Fahn & Williams criteria	Selection: Good Detection: Good Attrition: Good Reporting: Good
Schönenberg et al. (2015), Germany	Cross-sectional	PNES (Current symptoms)	15; 80%	32.34 (11.86)	15; 80%	32.2 (11.63)	Age, gender, years of education	TAS-20	Video-EEG, MINI	Selection: Good Detection: Good Attrition: Good Reporting: Good
Schulz et al. (2015), Germany	Cross-sectional	DPD (Chronic cases, current symptoms)	23; 52%	26.8 (6.5)	24; 54%	26.4 (2.0)	Age, gender	TAS-20 (DPD: 22 completed, Control: 23 completed)	SCID-D, SCID-V, ICD-10	Selection: Fair Detection: Good Attrition: Good Reporting: Good
Simeon et al.^31^ (2009), USA	Cross-sectional	DPD (Current symptoms)	46; 50.0%	30.59 (10.28)	35; 45.7%	31.94 (11.43)	Gender	TAS-20 (DPD: 33 completed, Control: 31 completed)	SCID-D, SCID-IV	Selection: Good Detection: Good Attrition: Good Reporting: Good
Sojka et al. (2019), Czech Republic	Cross-sectional	FMD (Chronic cases, current symptoms)	15; 73%	39.73 (16.54)	15; 73%	40.27 (15.88)	Age, gender, education	TAS-20	Fahn & Williams criteria	Selection: Good Detection: Good Attrition: Good Reporting: Fair
Urbanek et al.^68^ (2014), UK	Cross-sectional	PNES (Outpatients, current symptoms)	56; 64%	39.2 (13.6)	88; 70%	27.2 (9.3)	Gender, ethnicity	TAS-20	EEG or video-EEG & clinical assessment	Selection: Good Detection: Good Attrition: Good Reporting: Good
van Dijl et al. (2024), The Netherlands	Cross-sectional	FND (Current symptoms)	31; 54.8%	42.7 (14.8)	33; 63.6%	45.1 (16.2)	Age, gender, educational level	BVAQ	Clinical history, neurologic examination	Selection: Good Detection: Good Attrition: Good Reporting: Fair

*BVAQ* Bermond–Vorst Alexithymia Questionnaire, *CASH* Comprehensive Assessment of Symptoms and History, *CD* conversion disorder, *DIGS* Diagnostic Interview for Genetic Studies, *DPD* depersonalisation disorder, *DSM* Diagnostic and Statistical Manual of Mental Disorders, *EEG* electroencephalography, *FMD/FMS* functional motor disorder/symptoms, *FND* functional neurological disorder, *ICD-10* International Classification of Diseases-10, *MINI* Mini-International Neuropsychiatric Interview, *PNES* psychogenic non-epileptic seizure, *SCAN* Schedules for Clinical Assessment in Neuropsychiatry, *SCID* The Structured Clinical Interview for DSM, *SCID-D* The Structured Clinical Interview for Dissociative Disorders, *SSDs* schizophrenic spectrum disorders, *SZ* schizophrenia, *SZA* schizoaffective disorder, *TAS-20* Toronto Alexithymia Scale-20. The reference list of the included studies is shown in Supplementary Material S3.

#### Quality assessment

Quality appraisal is summarised in Table 1a. For selection bias, all but one study^57^ verified patient diagnoses with a structured clinical interview (e.g., Structured Clinical Interview for DSM [SCID], Mini International Neuropsychiatric Interview [MINI], Internal Classification of Disease [ICD]). Twenty-six papers described the recruitment procedure of patient samples, and 22 screened healthy controls for psychiatric illness, neurological injury, learning disability, or substance misuse. Seventeen also excluded those with a family history of psychiatric or psychotic disorders. Most studies (*k* = 26) matched groups on key demographic variables (e.g., age, gender, education) or controlled for unmatched variables in analyses. Overall, 21 studies were rated ‘Good’, four ‘Fair’ because of incomplete eligibility or screening information, and two ‘Poor’ due to missing details on recruitment and diagnostic assessment.

Detection bias was generally low. One study^58^ was rated ‘Fair’ for using combined samples from earlier work, which may have introduced procedural inconsistencies. Three others were rated ‘Fair’ because they failed to describe testing procedures in sufficient detail. Attrition bias was minimal: all studies had complete outcome data from recruited samples, and 11 explicitly reported how they dealt with missing values or outliers. For reporting bias, seven studies were rated ‘Fair’ due to unavailable alexithymia subscores.

#### Syntheses of results

**Fig. 2:**
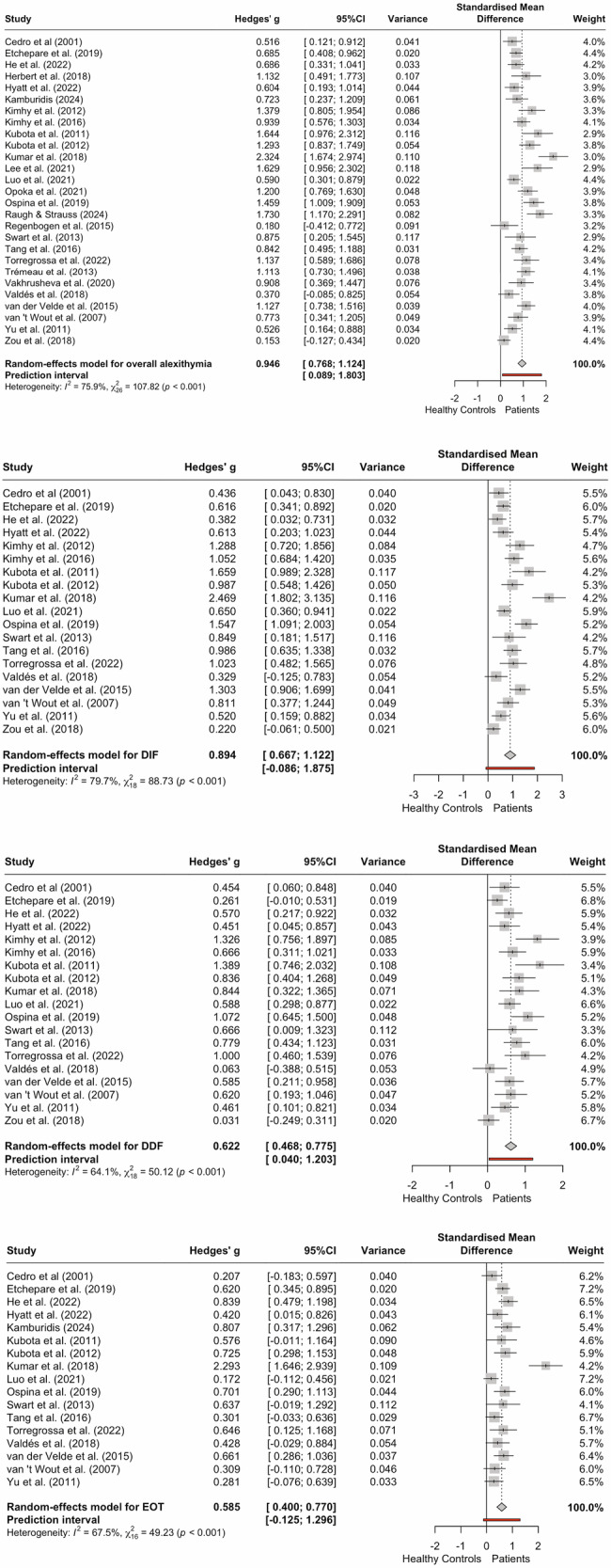
Meta-analysis 1—comparisons of alexithymia between patients with schizophrenia spectrum disorders and healthy controls. DIF difficulty identifying feelings, DDF difficulty describing feelings, EOT externally oriented thinking.

Figure 2 displays the forest plots for Meta-analysis 1, showing that patients with SSDs scored higher than controls on overall alexithymia and on each facet. As detailed in Table 2, aggregated Hedges’ *g* values were large for overall alexithymia and DIF and moderate for DDF and EOT, with substantial heterogeneity across all outcomes (Table 3).

**Table 2:** Effect size estimates for group comparison on alexithymia.

	Meta-analysis 1 (SSDs vs. HC)				Meta-analysis 2 (DDs vs. HC)				Moderator analysis	
	*k*	Hedges’ *g* (95%CI)	z	*p-*value	*k*	Hedges’ g (95%CI)	z	*p-*value	*Q*(*df)*	*p*-value
Overall	27	0.946 (0.768–1.124)	10.410	<0.001	30	1.164 (0.974–1.353)	12.030	<0.001	2.690 (1)	0.101
DIF	19	0.894 (0.667–1.122)	7.712	<0.001	19	1.311 (0.991–1.632)	8.018	<0.001	4.324 (1)	0.038
DDF	19	0.622 (0.468–0.775)	7.957	<0.001	19	0.954 (0.707–1.202)	7.548	<0.001	5.014 (1)	0.025
EOT	17	0.585 (0.400–0.770)	6.195	<0.001	19	0.427 (0.163–0.690)	3.171	0.002	0.932 (1)	0.335
*After removal of extreme effects*										
Overall	24	0.896 (0.755–1.036)	12.492	<0.001	28	1.162 (0.991–1.334)	13.282	<0.001	5.556 (1)	0.018
DIF	18	0.814 (0.628–1.000)	8.574	<0.001	18	1.240 (0.943–1.537)	8.175	<0.001	5.672 (1)	0.017
DDF	18	0.654 (0.517–0.792)	9.321	<0.001	19	0.954 (0.707–1.202)	7.548	<0.001	4.310 (1)	0.038
EOT	16	0.497 (0.377–0.617)	8.123	<0.001	17	0.276 (0.107–0.445)	3.203	0.001	4.370 (1)	0.037

*DIF* difficulty identifying feelings, *DDF* difficulty describing feelings, *EOT* externally oriented thinking, *DDs* dissociative disorders, *HC* healthy controls, *SSDs* schizophrenia spectrum disorders.

**Table 3:** Summary statistics for heterogeneity analyses.

	Meta-analysis 1 (SSDs vs. HC)			Meta-analysis 2 (DDs vs. HC)		
	*Q* (*df*)	*p*-value	*I*^2^ (%)	*Q* (*df*)	*p*-value	*I*^2^ (%)
Overall	107.817 (26)	<0.001	75.885	89.506 (29)	<0.001	67.600
DIF	88.726 (18)	<0.001	79.713	92.091 (18)	<0.001	80.454
DDF	50.122 (18)	<0.001	64.088	65.613 (18)	<0.001	72.566
EOT	49.226 (16)	<0.001	67.497	67.758 (18)	<0.001	73.435
*After removal of extreme effects*						
Overall	54.956 (23)	<0.001	58.149	63.058 (27)	<0.001	57.182
DIF	63.324 (17)	<0.001	73.154	81.296 (17)	<0.001	79.089
DDF	35.049 (17)	0.006	51.497	65.613 (18)	<0.001	72.566
EOT	19.941 (15)	0.174	24.776	25.370 (16)	0.064	36.932

*DIF* difficulty identifying feelings, *DDF* difficulty describing feelings, *EOT* externally oriented thinking, *DDs* dissociative disorders, *HC* healthy controls, *SSDs* schizophrenia spectrum disorders.

Pairwise tests indicated no significant difference in effect sizes between DDF and EOT (z = 0.302, *p* = 0.763). The DIF effect was marginally larger than DDF (z = 1.986, *p* = 0.052) and was significantly larger than EOT (z = 2.063, *p* = 0.039).

#### Publication bias

Supplementary Fig. 1 shows the funnel plots for Meta-analysis 1 (see Supplementary Material S4). Both Egger’s regression and the Begg-Mazumdar rank correlation were significant, indicating plot asymmetry and suggesting possible publication bias (Table 4).

**Table 4:** Summary statistics for risk of bias.

	Begg–Mazumdar test			Egger’s regression test			
	*Tau*	z	*p*-value	*b*	SE	*t*-value	*p*-value
Meta-analysis 1 (SSDs vs. HC)							
Overall	0.390	2.856	0.004	4.660	1.202	3.878	<0.001
DIF	0.439	2.624	0.009	5.484	1.568	3.496	0.003
DDF	0.427	2.554	0.011	4.406	1.239	3.557	0.002
EOT	0.309	1.730	0.084	3.673	1.618	2.270	0.038
Meta-analysis 2 (DDs vs. HC)							
Overall	−0.016	-0.125	0.901	−0.013	1.365	−0.009	0.993
DIF	0.170	1.015	0.310	2.384	1.807	1.320	0.204
DDF	0.029	0.175	0.861	0.218	1.742	0.125	0.902
EOT	0.170	1.015	0.310	0.587	1.661	0.354	0.728

*DIF* difficulty identifying feelings, *DDF* difficulty describing feelings, *EOT* externally oriented thinking, *DDs* dissociative disorders, *HC* healthy controls, *SSDs* schizophrenia spectrum disorders.

#### Sensitivity analysis

Effect sizes were not significantly associated with measurement instrument (*p*s > 0.050). Three studies with extreme effect sizes^59–61^ were excluded from the overall alexithymia analysis; Kumar et al.^59^ was removed from the DIF and EOT analyses, and Zou et al.^61^ from the DDF analysis. After exclusion, aggregated effects for every outcome remained significant (Table 2). The pairwise difference between DIF and DDF was no longer significant, and heterogeneity for EOT fell to a non-significant level (Table 3).

#### Subgroup analysis for SSDs

Subgroup analysis revealed that studies focusing solely on schizophrenia (*k* = 19) and those combining multiple SSD diagnoses (*k* = 8) were not significantly different in alexithymia effect sizes (*ps* > 0.050).

### Meta-analysis 2 results

Meta-analysis 2 included 30 studies comparing patients with any DDs against healthy controls, comprising 1638 participants (842 patients and 796 healthy controls). Demartini et al.^62^ reported separate PNES and FMD samples, both classified as conversion disorder; their means and SDs were pooled for effect size calculation. In total, 30 effect sizes were included in the meta-analysis of overall alexithymia. Subscale reporting was less consistent, with the DIF, DDF, and EOT analyses each including 19 effect sizes.

#### Study Characteristics

Table 1b outlines the study characteristics. Diagnoses were depersonalisation disorder (*k* = 5), conversion disorder (*k* = 7), PNES (*k* = 10), and FMD (*k* = 8). All studies recruited patients with recurrent symptoms; 25 studies excluded participants with comorbid or lifetime psychotic disorders, post‑traumatic stress disorder, or organic neurological conditions. Healthy controls were screened in 23 studies to confirm the absence of psychiatric diagnoses; one study also excluded controls with a family history of psychosis. Alexithymia was assessed predominantly with the TAS-20 (*k* = 29), with one study using the BVAQ.

#### Quality assessment

Quality appraisal is summarised in Table 1b. Most studies minimised selection bias: diagnoses were verified with structured clinical interviews (*k* = 19) and/or established clinical guidelines for functional neurologic symptoms (*k* = 20). FMD cases satisfied the Fahn-Williams’ criteria^63^ at a ‘clinically definite’ level (*k* = 9), while PNES cases were confirmed using LaFrance et al.’s criteria^64^ at ‘clinically documented’ or ‘clinically established’ levels, corroborated by electroencephalogram (EEG) and/or video-EEG recordings (*k* = 10). Key demographic variables (e.g., age, gender, education) were either group-matched or controlled as appropriate in all studies. Seven studies were rated ‘Fair’ and two ‘Poor’ because of limited psychiatric screening of controls or insufficient detail on eligibility and recruitment procedures.

For detection bias, all included studies were rated ‘Good’ as cases and controls underwent identical assessment procedures. Attrition bias was limited: 15 papers detailed how they handled missing or excluded data arising from incomplete responses, eligibility breaches, dropouts, non-response, outliers, or technical issues with neuroimaging. Eleven studies were rated ‘Fair’ on reporting bias for omitting alexithymia subscale scores.

#### Synthesis of results

**Fig. 3:**
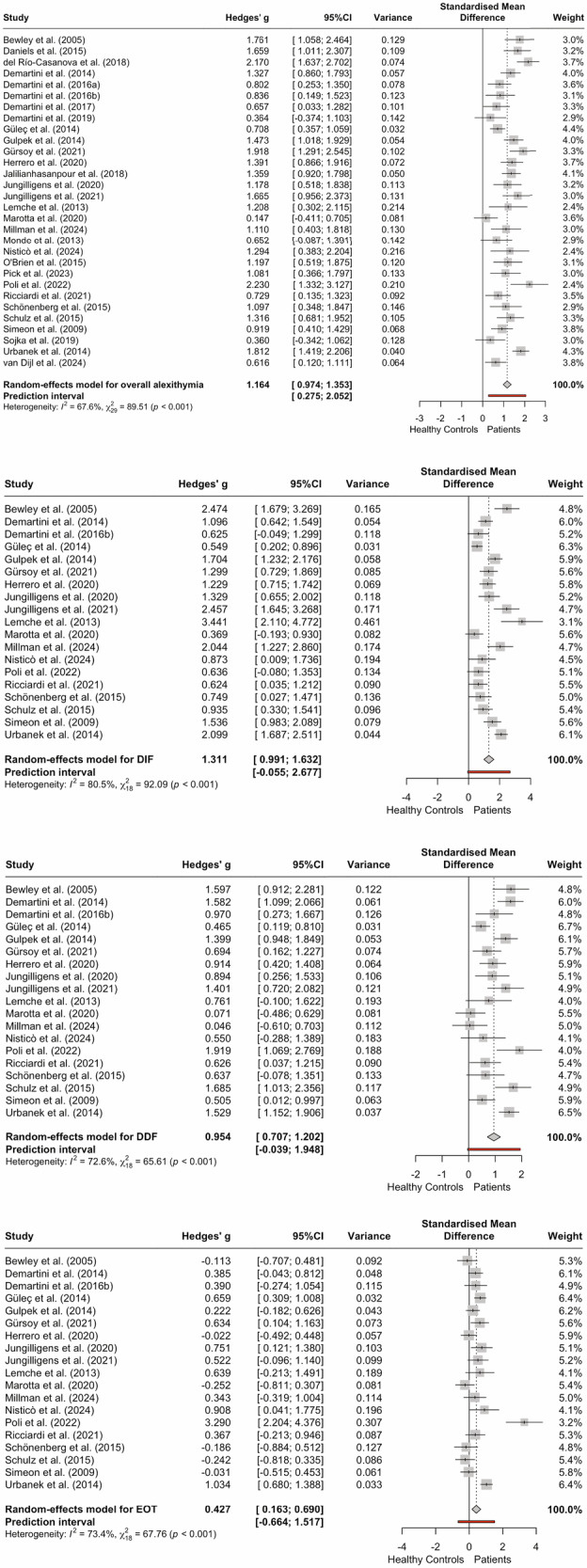
Meta-analysis 2—comparisons of alexithymia between patients with dissociative disorders and healthy controls. DIF difficulty identifying feelings, DDF difficulty describing feelings, EOT externally oriented thinking.

Figure 3 presents the forest plots for Meta‑analysis 2, showing that patients with DDs scored higher than controls on overall alexithymia and on each facet. Table 2 lists the aggregated Hedges’ *g* values, which were large for overall alexithymia, DIF, and DDF, and small-to-moderate for EOT. Heterogeneity ranged from moderate to high (Table 3).

Pairwise tests showed that DIF and DDF produced comparable effects (z = 1.725, *p* = 0.085). Both DIF (z = 4.175, *p* < 0.001) and DDF (z = 2.857, *p* = 0.004) showed significantly larger effects than EOT.

#### Publication bias

Supplementary Fig. 2 presents the funnel plots for Meta-analysis 2 (see Supplementary Material S5). Both the Begg-Mazumdar rank test and Egger’s regression found no evidence of asymmetry (Table 4).

#### Sensitivity analysis

Subgroup analysis by measurement instrument was not feasible, as only one study used the BVAQ. Two studies^65,66^ with extreme effects were removed from the overall alexithymia analysis, one^67^ from the DIF analysis, and two^32,68^ from the EOT analysis. Excluding these outliers did not alter the significance of any aggregated effects (Table 2) or the pattern of pairwise comparisons, and heterogeneity for EOT fell to a non-significant level (Table 3).

#### Subgroup analysis for DDs

Five depersonalisation studies formed the detachment subgroup and the other 25 studies (conversion disorder, PNES, FMD) formed the compartmentalisation subgroup. Because only three detachment papers reported subscale data, subgroup analysis was only performed on overall alexithymia. The aggregated effect did not differ between detachment and compartmentalisation subgroups (*p* = 0.926).

### Moderator analyses by disorder clusters

Moderator analyses contrasted the two disorder clusters on overall alexithymia and its facets (Table 2). Prior to outlier removal, DDs produced larger effects than SSDs for DIF and DDF (*ps* < 0.050), while overall alexithymia (*p* = 0.101) and EOT (*p* = 0.335) did not differ. After excluding outliers, DDs surpassed SSDs on overall alexithymia (*p* = 0.018) and continued to show stronger DIF and DDF effects (*ps* < .050). In contrast, the effect for EOT became larger in SSDs than in DDs (*p* = 0.037).

## Discussion

We conducted two meta-analyses to synthesise and compare effect sizes for overall alexithymia and its facets in healthy individuals versus those with SSDs (Meta-analysis 1) and DDs (Meta-analysis 2). By isolating facet-level effects and by including a broader range of diagnoses within each cluster, the current review advances earlier syntheses^17,29^. Although heterogeneity across studies was substantial, aggregated effects for overall alexithymia and for each facet remained significant even after removing statistical outliers, underscoring the robustness of the findings.

The two meta-analyses showed that overall alexithymia was elevated in both SSDs and DDs relative to healthy controls, with the large aggregated effects in both patient groups indicating a pervasive and clinically meaningful deficit, supporting alexithymia as a transdiagnostic correlate. In addition, facet-level patterns diverged across disorder clusters: in SSDs, DIF emerged to be the most marked deficit, followed by moderate elevations in DDF and EOT, whereas in DDs, both DIF and DDF showed large effects and EOT was only mildly increased compared to controls. Moderator analyses further indicated that elevations in DIF and DDF were significantly greater in DDs than in SSDs, both before and after excluding studies with extreme effect sizes, whereas EOT became more prominent in SSDs only after those exclusions. The facet-level differentiation implies that alexithymia should not be treated as a unitary construct when examining its role in SSDs versus DDs, and that disorder-specific facet profiles may index distinct patterns of emotional processing deficits.

The larger DIF and DDF effects observed in DDs may suggest that dissociation (i.e., disintegration of thoughts, feelings, and sensations) intensifies the split between bodily arousal, emotion labelling, and appraisal, whose disintegration or undifferentiation is at the core of alexithymia^69^. When individuals cannot recognise or articulate the triggers of their emotions, bodily sensations may become untethered from coherent emotional narratives, undermining emotion monitoring and regulation. Lynn et al.^70^ argue that this would render emotion unpredictable and abrupt, interfering with the continuity of emotional experience and its interplay with behaviours and cognitions, hence potentially fostering the sense of unreality and psychological numbness in dissociation-prone individuals. Alternatively, DIF and DDF are particularly associated with heightened negative affect^71^, a mechanism that may be especially relevant in DDs, where dissociation is thought to be triggered under conditions of hyperarousal and is closely tied to emotion dysregulation^72^.

After outlier removal, EOT was the only facet that showed a larger effect in SSDs than in DDs. Elevated EOT reflects an outward attentional bias or, as Vorst and Bermond^4^ argued, a limited capacity to analyse one’s own emotions. This bias could weaken the normal linking of emotion to its triggers and functions. Patients with schizophrenia, for example, show intact momentary emotional reactivity in the laboratory yet report diminished anticipatory pleasure and struggle to translate positive experiences into motivation^14^. Higher EOT may widen this gap by limiting introspection and the continuous updating of emotion schemas that bind imagery and action to felt emotions^69^, sustaining SSD symptoms such as anhedonia or social withdrawal. Nevertheless, the finding of a larger EOT effect size was only significant when outlier studies were removed, indicating that the result is sensitive to specific influential studies and should be interpreted cautiously, particularly given potential publication bias in the SSD literature, which may inflate effect sizes and underscores the need for replication.

Subgroup analyses found no differences in alexithymia effect sizes between studies limited to schizophrenia and those including other SSD diagnoses. The moderate-to-high heterogeneity in SSD effect sizes suggests that variability within the SSD spectrum may be influenced by factors beyond diagnoses. For instance, alexithymia may be especially elevated in patients with prominent negative symptoms^18^, yet the available data did not allow separate estimates for high- versus low-negative-symptom groups or evaluation of alexithymia’s relation to negative versus positive psychotic symptoms.

Likewise, subgroup analysis showed no difference in overall alexithymia between detachment and compartmentalisation dissociation. This null finding may reflect limited statistical power in the smaller detachment subgroup (*k* = 5) or the sizable heterogeneity observed across studies, suggesting variability in alexithymia levels or facets across the DD spectrum. Whether this absence of difference between detachment and compartmentalisation reflects a purely statistical artefact or a shared underlying ‘dissociative syndrome’^24^ that links both presentations to alexithymia remains unclear and warrants further investigation.

Interpretation of these findings should consider several limitations, particularly in light of the high between-study heterogeneity. First, effects of alexithymia were contrasted against non-psychiatric controls within each diagnostic group, not through a direct comparison between the patient samples. Second, gender distributions differed between SSD and DD studies, with more men in SSD samples and more women in DD samples. As alexithymia may be more prevalent in men^73^, this imbalance could influence mean alexithymia levels and complicate direct comparisons. Third, alexithymia scores were derived from self-report instruments, which depend on emotional insight that alexithymic individuals may lack^74^, even though self-reports correlate satisfactorily with observer ratings^75^. Fourth, while alexithymia overlaps with the construct emotional awareness, the latter was not examined in the current syntheses; further clarification of how these distinct aspects of emotional functioning^76^ operate in SSDs and DDs is needed. Fifth, although studies excluded comorbidity, dissociative symptoms frequently appear in SSDs and psychotic symptoms in DDs^39^, making it difficult to isolate the effects of co-occurring symptoms on alexithymia. Future studies should also consider additional clinical correlates shared by SSDs and DDs that may confound associations with alexithymia and emotional functioning, such as gender distribution, trauma history, and medication effects. Finally, the current cross-sectional evidence precludes causal inference. Although alexithymia appears independent of illness duration and antipsychotic treatment^77,78^, longitudinal, intervention, and experimental studies are needed to clarify its developmental course and dynamic (e.g., trait versus state) role across SSDs and DDs.

Despite these limitations, the divergent facet profiles between SSDs and DDs offer a more nuanced account of their differential emotional dysfunction and highlight potential psychotherapeutic targets not typically addressed in standard care. Since each facet may map onto multiple cognitive processes (e.g., attention, appraisal, memory, language)^79^, effective interventions may need to combine strategies^80^, such as attentional bias modification, interoceptive awareness, and emotional differentiation and expression^81^, tailored to the distinct facet profiles of SSDs and DDs.

## Conclusion

This study synthesised data of alexithymia severity in SSDs and DDs compared to healthy populations and report their respective facet profiles. In SSDs, difficulty identifying feelings emerged as the primary deficit, with smaller yet meaningful elevations in difficulty describing feelings and externally oriented thinking. In DDs, patients struggled with both identifying and describing feelings, whereas their externally oriented thinking was only modestly raised. Comparing the two disorder clusters reveals that DDs present greater deficits in identifying and describing feelings, whereas SSDs display a stronger bias toward externally oriented thinking. These distinct profiles underscore the need to move beyond global alexithymia scores and instead target facet-specific deficits when introducing emotion-focused interventions, alongside the usual symptom-focused approach to provide more comprehensive care for patients with SSDs and DDs.

## Supplementary information

Supplementary Materials S1–5

## Data Availability

This systematic review was based on data from previously published articles, no original data were collected. All data generated or analysed during this study are included in this article or the supplement. Further inquiries can be directed to the corresponding author.
