# Supplementary material for: Alexithymia in schizophrenia spectrum disorders and dissociative disorders: two meta-analytic reviews
Source: Schizophrenia (Heidelb). 2026 May 14;12(1):66. doi: 10.1038/s41537-026-00765-8 (PMC13415523; doi:10.1038/s41537-026-00765-8)

**Supplementary Materials**

**Alexithymia in schizophrenia spectrum disorders and dissociative disorders: Two meta-analytic reviews**

# **Supplementary Material S1**

**PRISMA 2020 Checklist**

| **Section and Topic** | **Item #** | **Checklist item** | **Location where item is reported** |
| --- | --- | --- | --- |
| **TITLE** | | |  |
| Title | 1 | Identify the report as a systematic review. | P.1 |
| **ABSTRACT** | | |  |
| Abstract | 2 | See the PRISMA 2020 for Abstracts checklist. | P.2 |
| **INTRODUCTION** | | |  |
| Rationale | 3 | Describe the rationale for the review in the context of existing knowledge. | P.3-5 |
| Objectives | 4 | Provide an explicit statement of the objective(s) or question(s) the review addresses. | P.5 |
| **METHODS** | | |  |
| Eligibility criteria | 5 | Specify the inclusion and exclusion criteria for the review and how studies were grouped for the syntheses. | P.6 |
| Information sources | 6 | Specify all databases, registers, websites, organisations, reference lists and other sources searched or consulted to identify studies. Specify the date when each source was last searched or consulted. | P.6-7 |
| Search strategy | 7 | Present the full search strategies for all databases, registers and websites, including any filters and limits used. | P.6-7 |
| Selection process | 8 | Specify the methods used to decide whether a study met the inclusion criteria of the review, including how many reviewers screened each record and each report retrieved, whether they worked independently, and if applicable, details of automation tools used in the process. | P.6-7 |
| Data collection process | 9 | Specify the methods used to collect data from reports, including how many reviewers collected data from each report, whether they worked independently, any processes for obtaining or confirming data from study investigators, and if applicable, details of automation tools used in the process. | P.7 |
| Data items | 10a | List and define all outcomes for which data were sought. Specify whether all results that were compatible with each outcome domain in each study were sought (e.g. for all measures, time points, analyses), and if not, the methods used to decide which results to collect. | P.7 |
|  | 10b | List and define all other variables for which data were sought (e.g. participant and intervention characteristics, funding sources). Describe any assumptions made about any missing or unclear information. | P.7 |
| Study risk of bias assessment | 11 | Specify the methods used to assess risk of bias in the included studies, including details of the tool(s) used, how many reviewers assessed each study and whether they worked independently, and if applicable, details of automation tools used in the process. | P.7-8, Suppl material S2 |
| Effect measures | 12 | Specify for each outcome the effect measure(s) (e.g. risk ratio, mean difference) used in the synthesis or presentation of results. | P.8 |
| Synthesis methods | 13a | Describe the processes used to decide which studies were eligible for each synthesis (e.g. tabulating the study intervention characteristics and comparing against the planned groups for each synthesis (item #5)). | P.7-8 |
|  | 13b | Describe any methods required to prepare the data for presentation or synthesis, such as handling of missing summary statistics, or data conversions. | P.8 |
|  | 13c | Describe any methods used to tabulate or visually display results of individual studies and syntheses. | P.10, 13 |
|  | 13d | Describe any methods used to synthesize results and provide a rationale for the choice(s). If meta-analysis was performed, describe the model(s), method(s) to identify the presence and extent of statistical heterogeneity, and software package(s) used. | P.8 |
|  | 13e | Describe any methods used to explore possible causes of heterogeneity among study results (e.g. subgroup analysis, meta-regression). | P.8-9 |
|  | 13f | Describe any sensitivity analyses conducted to assess robustness of the synthesized results. | P.8 |
| Reporting bias assessment | 14 | Describe any methods used to assess risk of bias due to missing results in a synthesis (arising from reporting biases). | P.8 |
| Certainty assessment | 15 | Describe any methods used to assess certainty (or confidence) in the body of evidence for an outcome. | N/A |
| **RESULTS** | | |  |
| Study selection | 16a | Describe the results of the search and selection process, from the number of records identified in the search to the number of studies included in the review, ideally using a flow diagram. | Figure 1 |
|  | 16b | Cite studies that might appear to meet the inclusion criteria, but which were excluded, and explain why they were excluded. | N/A |
| Study characteristics | 17 | Cite each included study and present its characteristics. | Table 1 |
| Risk of bias in studies | 18 | Present assessments of risk of bias for each included study. | Table 1 |
| Results of individual studies | 19 | For all outcomes, present, for each study: (a) summary statistics for each group (where appropriate) and (b) an effect estimate and its precision (e.g. confidence/credible interval), ideally using structured tables or plots. | Figures 2, 3 |
| Results of syntheses | 20a | For each synthesis, briefly summarise the characteristics and risk of bias among contributing studies. | P.9-10, 12-13 |
|  | 20b | Present results of all statistical syntheses conducted. If meta-analysis was done, present for each the summary estimate and its precision (e.g. confidence/credible interval) and measures of statistical heterogeneity. If comparing groups, describe the direction of the effect. | Tables 2, 3 |
|  | 20c | Present results of all investigations of possible causes of heterogeneity among study results. | P.11, 13-14 |
|  | 20d | Present results of all sensitivity analyses conducted to assess the robustness of the synthesized results. | P.11, 13-14 |
| Reporting biases | 21 | Present assessments of risk of bias due to missing results (arising from reporting biases) for each synthesis assessed. | Table 4, Suppl material S4-5 |
| Certainty of evidence | 22 | Present assessments of certainty (or confidence) in the body of evidence for each outcome assessed. | N/A |
| **DISCUSSION** | | |  |
| Discussion | 23a | Provide a general interpretation of the results in the context of other evidence. | P.14-16 |
|  | 23b | Discuss any limitations of the evidence included in the review. | P.17 |
|  | 23c | Discuss any limitations of the review processes used. | P.17 |
|  | 23d | Discuss implications of the results for practice, policy, and future research. | P.17-18 |
| **OTHER INFORMATION** | | |  |
| Registration and protocol | 24a | Provide registration information for the review, including register name and registration number, or state that the review was not registered. | P.5 |
|  | 24b | Indicate where the review protocol can be accessed, or state that a protocol was not prepared. | P.5 |
|  | 24c | Describe and explain any amendments to information provided at registration or in the protocol. | P.5-6 |
| Support | 25 | Describe sources of financial or non-financial support for the review, and the role of the funders or sponsors in the review. | P.19 |
| Competing interests | 26 | Declare any competing interests of review authors. |  |
| Availability of data, code and other materials | 27 | Report which of the following are publicly available and where they can be found: template data collection forms; data extracted from included studies; data used for all analyses; analytic code; any other materials used in the review. |  |

# **Supplementary Material S2**

**AHRQ risk of bias criteria**

General instructions: Rate each criterion as 'Good' (low risk of bias), 'Fair', 'Poor' (high risk of bias). Factors to consider when rating each criterion are listed below.

| **Selection bias** | - Clear description of recruitment strategy for the case and control groups. - Clear description of inclusion and exclusion criteria. - Consistency of exclusion criteria between clinical and control groups. - Comparability of clinical and control groups in terms of demographics (comprehensive information provided? Matched/controlled for demographics, i.e. age, gender, ethnicity, education level, etc.?). - Validity of method for ascertaining clinical diagnosis (use of formal diagnostic interviews instead of symptom rating scales). - Validity of method and screening procedure for control group. - Appropriateness of adjustment for important confounding variables. |
| --- | --- |
| **Detection bias** | - Blindness of assessors to participants' clinical status. - Validity of assessment for alexithymia. - Consistency of measure implementation and testing procedure for all participants. |
| **Attrition bias** | - Completeness of outcome data. - Appropriate handling of missing data. - Systematic difference between clinical and control groups in terms of data completeness. - Lack of systematic difference between completers and non-completers in terms of outcome data. |
| **Reporting bias** | - Completeness of outcome reporting (were all the potential outcomes pre-specified by the authors? Were all prespecified outcomes reported?) - Availability of total and subscale scores of alexithymia measures - Lack of conflict of interests |

# **Supplementary Material S3**

**Reference list for papers included in Meta-analysis 1 (*k* = 27)**

Cedro, A., Kokoszka, A. & Popiel, A. Alexithymia in schizophrenia: an exploratory study. *Psychol. Rep.***89**, 95–98. (2001).

Etchepare, A.*et al*. What are the specificities of social cognition in schizophrenia? A cluster-analytic study comparing schizophrenia with the general population. *Psychiatry Res.***272**, 369–379. <https://doi.org/10.1016/j.psychres.2018.12.042> (2019).

He, C.*et al*. Exploring the link between cognitive deficit, self-esteem, alexithymia, and depressive symptom of schizophrenia. *Brain Behav***12**, e2648. <https://doi.org/10.1002/brb3.2648> (2022).

Herbert, C., Hesse, K. & Wildgruber, D. Emotion and self in psychotic disorders: Behavioral evidence from an emotional evaluation task using verbal stimuli varying in emotional valence and self-reference. *J. Behav. Ther. Exp. Psychiatry***58**, 86–96. <https://doi.org/10.1016/j.jbtep.2017.09.003> (2018).

Hyatt, C. J.*et al*. Atypical dynamic functional network connectivity state engagement during social-emotional processing in schizophrenia and autism. *Cereb Cortex***32**, 3406–3422. <https://doi.org/10.1093/cercor/bhab423> (2022).

Kamburidis, J. A. Relationship of Alexithymia with Emotion Regulation Strategies and Mental Health in Schizophrenic Patients. *J Evid-Based Psychother***24**, 109–116. <https://doi.org/10.24193/jebp.2024.1.6> (2024).

Kimhy, D.*et al*. The impact of emotion awareness and regulation on social functioning in individuals at clinical high risk for psychosis. *Psychol. Med.***46**, 2907–2918. <https://doi.org/10.1017/S0033291716000490> (2016).

Kimhy, D.*et al*. Emotion awareness and regulation in individuals with schizophrenia: Implications for social functioning. *Psychiatry Res.***200**, 193–201. <https://doi.org/10.1016/j.psychres.2012.05.029> (2012).

Kubota, M.*et al*. Alexithymia and regional gray matter alterations in schizophrenia. *Neurosci. Res.***70**, 206–213. <https://doi.org/10.1016/j.neures.2011.01.019> (2011).

Kubota, M.*et al*. Alexithymia and reduced white matter integrity in schizophrenia: A diffusion tensor imaging study on impaired emotional self-awareness. *Schizophr. Res.***141**, 137–143. <https://doi.org/10.1016/j.schres.2012.08.026> (2012).

Kumar, S., Mohanty, S. & Gaur, A. Alexithymia in Schizophrenia: Association with Psychopathology, Emotional Intelligence and Cognitive Emotion Regulation. *Indian J Clin Psychol***45**, 42–50. (2018).

Lee, S. K.*et al*. The relationship between ambivalence, alexithymia, and salience network dysfunction in schizophrenia. *Psychiatry Res Neuroimaging***310**, 111271. <https://doi.org/10.1016/j.pscychresns.2021.111271> (2021).

Luo, H.*et al*. A bottom-up model of functional outcome in schizophrenia. *Sci Rep***11**, 7577. <https://doi.org/10.1038/s41598-021-87172-4> (2021).

Opoka, S. M., Sundag, J., Riehle, M. & Lincoln, T. M. Emotion-Regulation in Psychosis: Patients with Psychotic Disorders Apply Reappraisal Successfully. *Cogn Ther Res***45**, 31–45. <https://doi.org/10.1007/s10608-020-10163-8> (2021).

Ospina, L. H.*et al*. Alexithymia predicts poorer social and everyday functioning in schizophrenia and bipolar disorder. *Psychiatry Res.***273**, 218–226. <https://doi.org/10.1016/j.psychres.2019.01.033> (2019).

Raugh, I. M. & Strauss, G. P. Trait Mindfulness in Psychotic Disorders: Dimensions Predicting Symptoms, Cognition, and Functional Outcome. *Behav Therapy***55**, 55–67. <https://doi.org/10.1016/j.beth.2023.05.004> (2024).

Regenbogen, C.*et al*. Neural responses to dynamic multimodal stimuli and pathology-specific impairments of social cognition in schizophrenia and depression. *Br J Psychiatry***206**, 198–205. <https://doi.org/10.1192/bjp.bp.113.143040> (2015).

Swart, M.*et al*. Normal brain activation in schizophrenia patients during associative emotional learning. *Psychiatry Res Neuroimaging***214**, 269–276. <https://doi.org/10.1016/j.pscychresns.2013.08.008> (2013).

Tang, X. W.*et al*. Facial emotion recognition and alexithymia in Chinese male patients with deficit schizophrenia. *Psychiatry Res.***246**, 353–359. <https://doi.org/10.1016/j.psychres.2016.09.055> (2016).

Torregrossa, L. J., Amedy, A., Roig, J., Prada, A. & Park, S. Interoceptive functioning in schizophrenia and schizotypy. *Schizophr. Res.***239**, 151–159. <https://doi.org/10.1016/j.schres.2021.11.046> (2022).

Trémeau, F., Goldman, J., Antonius, D. & Javitt, D. C. Inpatients with schizophrenia report impaired situational motivation but intact global and social motivation. *Psychiatry Res.***210**, 43–49. <https://doi.org/10.1016/j.psychres.2013.05.031> (2013).

Vakhrusheva, J.*et al*. Lexical analysis of emotional responses to “real-world” experiences in individuals with schizophrenia. *Schizophr. Res.***216**, 272–278. <https://doi.org/10.1016/j.schres.2019.11.045> (2020).

Valdés, M.*et al*. Somatosensory amplification in schizophrenia is associated with preserved neuropsychological function. *Revista de Psiquiatría y Salud Mental***1**, 3–9. <https://doi.org/10.1016/S1888-9891(08)72510-2> (2008).

van 't Wout, M., Aleman, A., Bermond, B. & Kahn, R. S. No words for feelings: alexithymia in schizophrenia patients and first-degree relatives. *Compr. Psychiatry***48**, 27–33. <https://doi.org/10.1016/j.comppsych.2006.07.003> (2007).

Van Der Velde, J.*et al*. Cognitive alexithymia is associated with the degree of risk for psychosis. *PLoS ONE***10**, e0124803. <https://doi.org/10.1371/journal.pone.0124803> (2015).

Yu, S.*et al*. Alexithymia and personality disorder functioning styles in paranoid schizophrenia. *Psychopathology***44**, 371–378. <https://doi.org/10.1159/000325168> (2011).

Zou, Y.*et al*. Profiling of experiential pleasure, emotional regulation and emotion expression in patients with schizophrenia. *Schizophr. Res.***195**, 396–401. <https://doi.org/10.1016/j.schres.2017.08.048> (2018).

**Reference list for papers included in Meta-analysis 2 (*k* = 30)**

Bewley, J., Murphy, P. N., Mallows, J. & Baker, G. A. Does alexithymia differentiate between patients with nonepileptic seizures, patients with epilepsy, and nonpatient controls? *Epilepsy Behav***7**, 430–437. <https://doi.org/10.1016/j.yebeh.2005.06.006> (2005).

Daniels, J., Gaebler, M., Lamke, J. P. & Walter, H. Grey matter alterations in patients with depersonalization disorder: A voxel-based morphometry study. *J Psychiatry Neurosci***40**, 19–27. <https://doi.org/10.1503/jpn.130284> (2015).

del Río-Casanova, L.*et al*. The role of emotion dysregulation in Conversion Disorder. *Actas Esp. Psiquiatr.***46**, 92–103. (2018).

Demartini, B.*et al*. The truth about cognitive impairment in functional motor symptoms: An experimental deception study with the Guilty Knowledge Task. *J Clin Neurosci***64**, 174–179. <https://doi.org/10.1016/j.jocn.2019.03.005> (2019).

Demartini, B.*et al*. Psychogenic non-epileptic seizures and functional motor symptoms: A common phenomenology? *J. Neurol. Sci.***368**, 49–54. <https://doi.org/10.1016/j.jns.2016.06.045> (2016).

Demartini, B.*et al*. Anorexia nervosa and functional motor symptoms: Two faces of the same coin? *J. Neuropsychiatry Clin. Neurosci.***29**, 383–390. <https://doi.org/10.1176/appi.neuropsych.16080156> (2017).

Demartini, B.*et al*. The role of alexithymia in the development of functional motor symptoms (conversion disorder). *J Neurol Neurosurg Psychiatry***85**, 1132–1137. <https://doi.org/10.1136/jnnp-2013-307203> (2014).

Demartini, B., Ricciardi, L., Crucianelli, L., Fotopoulou, A. & Edwards, M. J. Sense of body ownership in patients affected by functional motor symptoms (conversion disorder). *Conscious. Cogn.***39**, 70–76. <https://doi.org/10.1016/j.concog.2015.11.005> (2016).

Gulpek, D., Kelemence Kaplan, F., Kesebir, S. & Bora, O. Alexithymia in patients with conversion disorder. *Nord J Psychiatr***68**, 300–305. <https://doi.org/10.3109/08039488.2013.814711> (2014).

Güleç, M. Y., Ýnanç, L., Yanartap, Ö, Üzer, A. & Güleç, H. Predictors of suicide in patients with conversion disorder. *Compr. Psychiatry***55**, 457–462. <https://doi.org/10.1016/j.comppsych.2013.10.009> (2014).

Gürsoy, S. C., Ergün, S., Midi, İ & Topçuoğlu, V. Theory of mind and its relationship with alexithymia and quality of life in patients with psychogenic nonepileptic seizures: Comparisons with generalised epilepsy and healthy controls. *Seizure***91**, 251–257. <https://doi.org/10.1016/j.seizure.2021.06.032> (2021).

Herrero, H.*et al*. Skin conductance response and emotional response in women with psychogenic non-epileptic seizures. *Seizure***81**, 123–131. <https://doi.org/10.1016/j.seizure.2020.07.028> (2020).

Jalilianhasanpour, R.*et al*. Resilience linked to personality dimensions, alexithymia and affective symptoms in motor functional neurological disorders. *J. Psychosom. Res.***107**, 55–61. <https://doi.org/10.1016/j.jpsychores.2018.02.005> (2018).

Jungilligens, J.*et al*. Microstructural integrity of affective neurocircuitry in patients with dissociative seizures is associated with emotional task performance, illness severity and trauma history. *Seizure***84**, 91–98. <https://doi.org/10.1016/j.seizure.2020.11.021> (2021).

Jungilligens, J.*et al*. Impaired emotional and behavioural awareness and control in patients with dissociative seizures. *Psychol. Med.*, 2731–2739. <https://doi.org/10.1017/S0033291719002861> (2019).

Lemche, E.*et al*. Interoceptive-reflective regions differentiate alexithymia traits in depersonalization disorder. *Psychiatry Res Neuroimaging***214**, 66–72. <https://doi.org/10.1016/j.pscychresns.2013.05.006> (2013).

Marotta, A.*et al*. Attentional avoidance of emotions in functional movement disorders. *J. Psychosom. Res.***133**, 110100. <https://doi.org/10.1016/j.jpsychores.2020.110100> (2020).

Millman, L. S. M.*et al*. Etiological Factors and Symptom Triggers in Functional Motor Symptoms and Functional Seizures: A Pilot Investigation. *J. Neuropsychiatry Clin. Neurosci.*, 350–357. <https://doi.org/10.1176/appi.neuropsych.20230103> (2024).

Monde, K. M., Ketay, S., Giesbrecht, T., Braun, A. & Simeon, D. Preliminary physiological evidence for impaired emotion regulation in depersonalization disorder. *Psychiatry Res.***209**, 235–238. <https://doi.org/10.1016/j.psychres.2013.02.020> (2013).

Nisticò, V.*et al*. Forearm bisection task suggests an alteration in body schema in patients with functional movement disorders (motor conversion disorders). *J. Psychosom. Res.***178**, 111610. <https://doi.org/10.1016/j.jpsychores.2024.111610> (2024).

O'Brien, F. M.*et al*. Psychiatric and neuropsychological profiles of people with psychogenic nonepileptic seizures. *Epilepsy Behav***43**, 39–45. <https://doi.org/10.1016/j.yebeh.2014.11.012> (2015).

Pick, S.*et al*. Objective and subjective neurocognitive functioning in functional motor symptoms and functional seizures: preliminary findings. *J Clin Exp Neuropsychol***45**, 970–987. <https://doi.org/10.1080/13803395.2023.2245110> (2023).

Poli, A.*et al*. Different dissociation and alexithymia domains specifically relate to patients with psychogenic non-epileptic seizures (PNES) and with PNES and comorbid epilepsy (PNES+EP). *J Affect Disord Rep***7**, 100296. <https://doi.org/10.1016/j.jadr.2021.100296> (2022).

Ricciardi, L.*et al*. Exploring three levels of interoception in people with functional motor disorders. *Parkinsonism Relat Disord***86**, 15–18. <https://doi.org/10.1016/j.parkreldis.2021.03.029> (2021).

Schulz, A.*et al*. Altered patterns of heartbeat-evoked potentials in depersonalization/derealization disorder: Neurophysiological evidence for impaired cortical representation of bodily signals. *Psychosom. Med.***77**, 506–516. <https://doi.org/10.1097/PSY.0000000000000195> (2015).

Schönenberg, M.*et al*. Theory of mind abilities in patients with psychogenic nonepileptic seizures. *Epilepsy Behav***53**, 20–24. <https://doi.org/10.1016/j.yebeh.2015.09.036> (2015).

Simeon, D., Giesbrecht, T., Knutelska, M., Smith, R. J. & Smith, L. M. Alexithymia, Absorption, and Cognitive Failures in Depersonalization Disorder. *J Nerv Ment Dis***197**, 492–498. <https://doi.org/10.1097/nmd.0b013e3181aaef6b> (2009).

Sojka, P.*et al*. Processing of emotions in functional movement disorder: An exploratory fMRI study. *Front Neurol***10**, 861. <https://doi.org/10.3389/fneur.2019.00861> (2019).

Urbanek, M., Harvey, M., McGowan, J. & Agrawal, N. Regulation of emotions in psychogenic nonepileptic seizures. *Epilepsy Behav***37**, 110–115. <https://doi.org/10.1016/j.yebeh.2014.06.004> (2014).

van Dijl, T. L.*et al*. Alexithymia and facial emotion recognition in patients with functional neurological disorder. *Clin. Neurol. Neurosurg.***237**, 108128. <https://doi.org/10.1016/j.clineuro.2024.108128> (2024).

# **Supplementary Material S4**

Supplementary Fig. 1. Funnel plots of publication bias analysis for Meta-analysis 1. DIF, difficulty identifying feelings; DDF, difficulty describing feelings; EOT, externally oriented thinking.


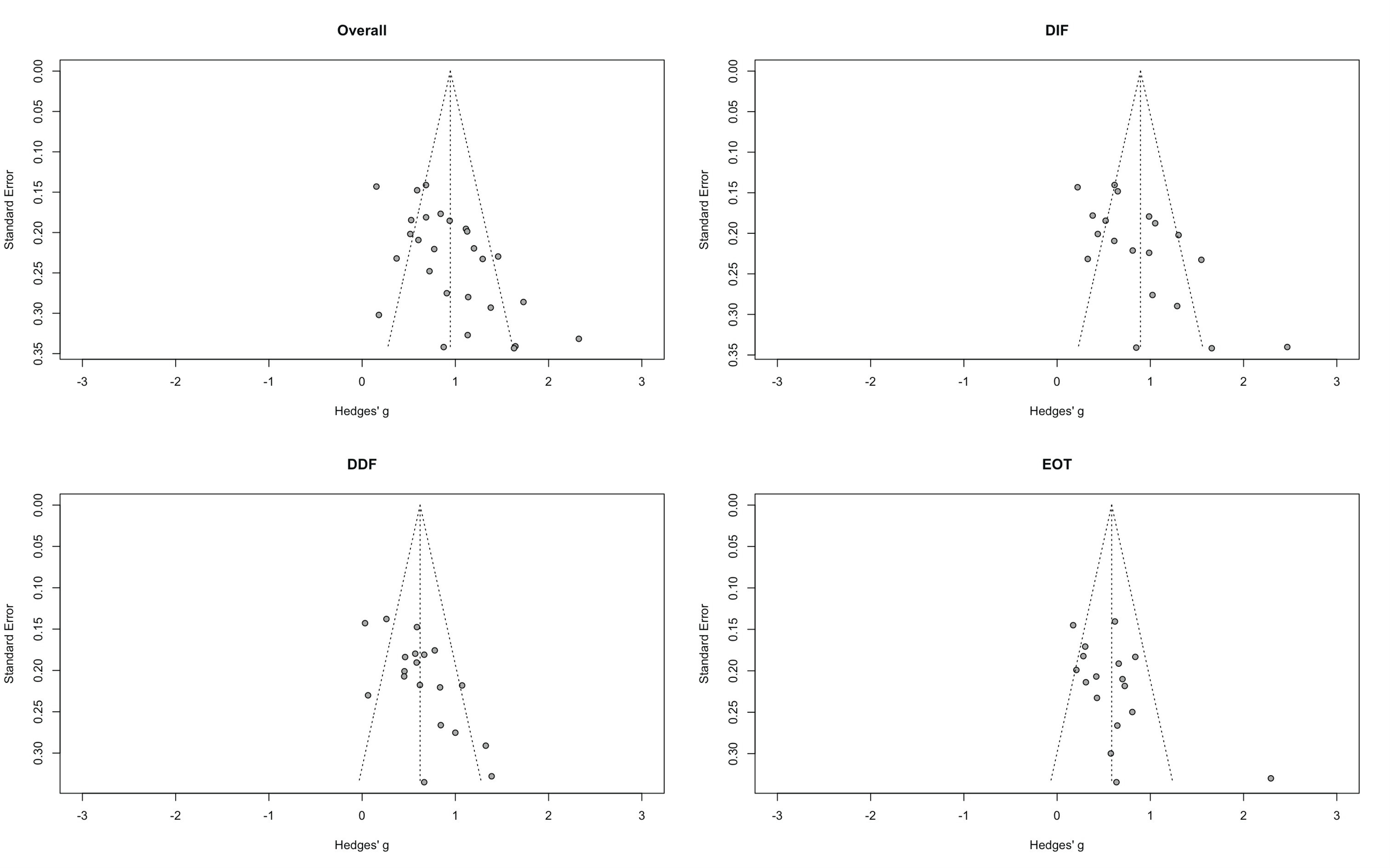


# **Supplementary Material S5**

Supplementary Fig. 2. Funnel plots of publication bias analysis for Meta-analysis 2. DIF, difficulty identifying feelings; DDF, difficulty describing feelings; EOT, externally oriented thinking.


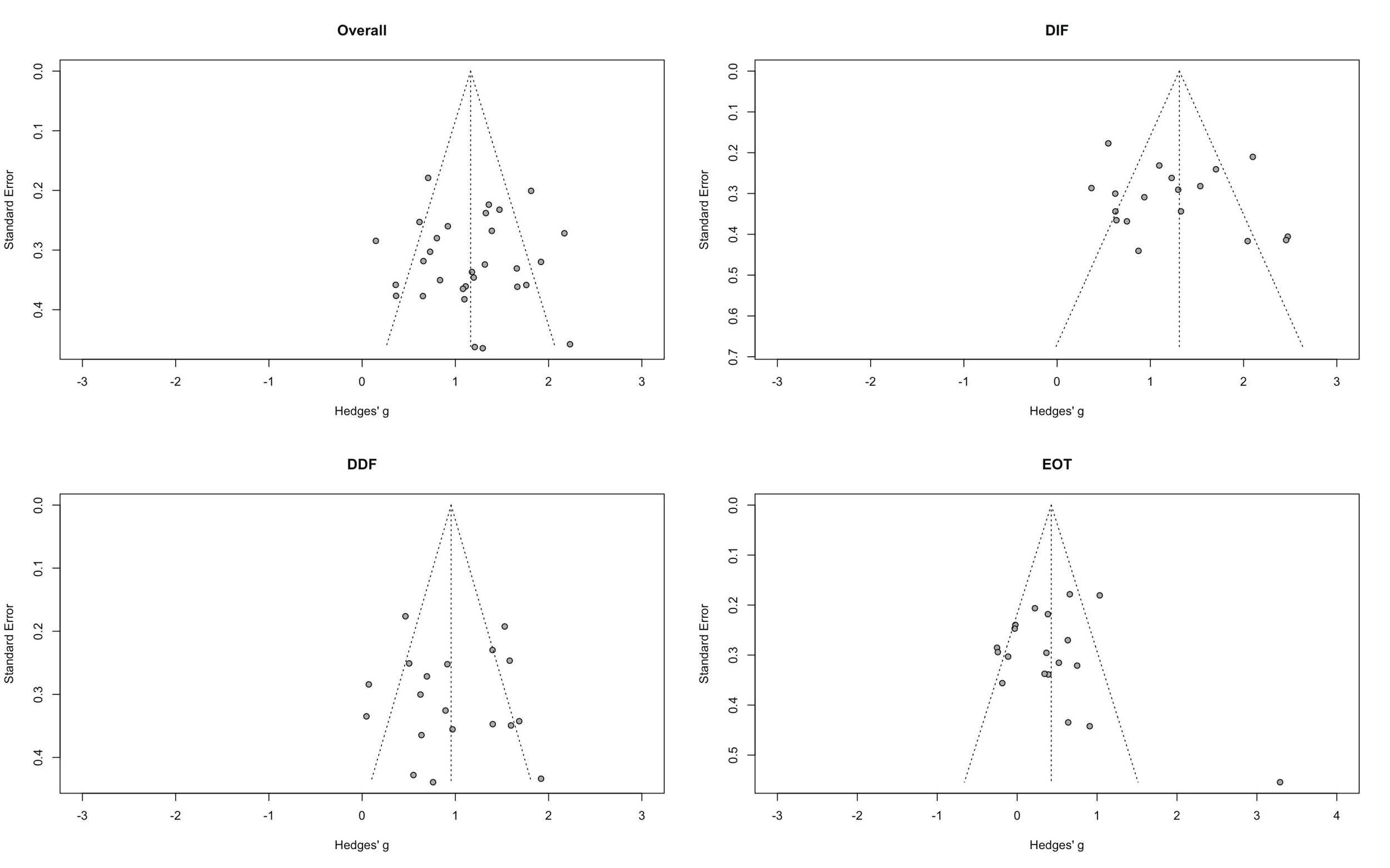

Supplement: Supplementary file 1 — Supplementary Materials S1–5 [file 41537_2026_765_MOESM1_ESM.docx]
